# Using an Analog Multifunctional Model to Develop Cognitive Skills in Dental Implants

**DOI:** 10.1155/ijod/6678271

**Published:** 2026-08-23

**Authors:** Morbeck dos Santos Leal, Ilana Dantas Neves, Atson Carlos de Souza Fernandes, Alena Ribeiro Alves Peixoto Medrado

**Affiliations:** ^1^ Dental Implantology Department, Bahiana School of Medicine and Public Health, Salvador, Bahia, Brazil, bahiana.edu.br; ^2^ Bio-interaction Department, Health and Sciences Institute of Federal University of Bahia, Salvador, Bahia, Brazil; ^3^ Life Sciences Department, University of the State of Bahia, Salvador, Bahia, Brazil

**Keywords:** dental implants, dentistry, higher education, learning, teaching methods

## Abstract

**Objective:**

To evaluate the effectiveness of the multifunctional analog model (MAM) as a teaching–learning tool for developing cognitive skills in dental implantology among undergraduate students.

**Materials and Methods:**

This randomized educational trial included 115 students allocated into three groups: group A (theoretical instruction), group B (theoretical instruction plus laboratory practice), and group C (laboratory practice only). Learning was assessed using a five‐item objective questionnaire administered before and after the intervention. Students’ perceptions were evaluated through two open‐ended questions.

**Results:**

The percentage of correct answers increased from 33.3% (SD 17.4) to 55.4% (SD 26.1) in group A, from 28.2% (SD 20.4) to 76.9% (SD 20.8) in group B, and from 23.8% (SD 18.2) to 77.3% (SD 17.7) in group C. Posttest performance was significantly higher in groups B and C compared with group A (*p* < 0.05), with no significant difference between groups B and C. Most participants (99.2%) preferred the applied methodology over traditional lectures, with integration of theory and practice identified as the most relevant aspect (31.9%).

**Conclusion:**

The MAM‐based methodology was significantly more effective than theoretical instruction alone in promoting cognitive learning. The integration of theory and practice was the key factor underlying its effectiveness.

## 1. Introduction

Dental implantology was recognized as a dental specialty in Brazil by the Federal Council of Dentistry (CFO) in 1990 owing to its growing benefits to the population. This recognition posed significant challenges for educators in integrating the discipline into the curricula of higher education institutions (HEIs), given its technical complexity and the lack of structured pedagogical methodologies for teaching this skill at the undergraduate level in dentistry [1, 2].

The National Curriculum Guidelines of 2002 were updated in 2018 under Opinion Number 803/2018 of the Higher Education Chamber of the National Council of Education establishing a student‐centered educational approach. These guidelines recommend replacing traditional lecture‐based teaching with the development and application of active learning methodologies (ALMs) to overcome the theory–practice dichotomy. The goal is to promote academic training that can be effectively applied to daily professional practice, particularly in healthcare settings [3–6]. This approach is grounded in Capra’s (1982) concept, which holds that students should be able to “learn to know,” “learn to do,” “learn to live together,” “learn to learn,” and “learn to be.” Since then, discussions have focused on ALMs and the strategies required for their effective implementation; however, this process remains complex and challenging.

One of the primary challenges is resistance from both faculty and students to leaving their “comfort zones,” often due to apprehension and insecurity, as they are accustomed to a traditional educational model based on the transmission of “ready‐made” knowledge [7, 8]. Additionally, implementing ALMs requires HEIs to invest more substantively in infrastructure, expand and train faculty, reduce curricular content, and decrease the number of students per class [9–12].

Nevertheless, with appropriate planning and creativity, existing preclinical laboratories in dental schools can be effectively utilized for this purpose. Simulated treatments using manikins or plaster models are essential for student training as they facilitate knowledge acquisition and retention while reducing insecurity when applying techniques in clinical settings. Furthermore, preclinical training that enhances cognitive and psychomotor skills may help minimize surgical–prosthetic complications in patients undergoing dental implant rehabilitation [13].

In this context, a reproducible, low‐cost educational tool designed to support undergraduate learning in implant dentistry, the multifunctional analog model (MAM), is proposed. This model consists of a plaster cast with edentulous areas that simulate clinical scenarios in which surgical–prosthetic concepts related to dental implants are addressed. It enables the direct application of knowledge by integrating theory and practice in an experiential, interactive, and collaborative learning environment.

This study was motivated by the need to develop new educational tools and/or ALMs in implant dentistry, given the scarcity of relevant studies identified in the literature. The primary objective was to evaluate the cognitive learning achieved by undergraduate dental students regarding the first surgical stage (FSS) of dental implant placement using the MAM and to compare it with the traditional method of theoretical instruction on the same topic. Additionally, the study aimed to assess students’ perceptions of the teaching–learning methodology associated with this educational tool.

## 2. Materials and Methods

This study was approved by the Research Ethics Committee of the Bahiana School of Medicine and Public Health (CAAE: 57642522.9.0000.5544; Approval Number: 5.470.074). Written informed consent was obtained from the patient for the use of anonymized clinical and imaging data in the development of educational materials and for scientific research. It was designed as a randomized educational trial involving 115 undergraduate dental students from four HEIs in the state of Bahia, Brazil, conducted between June 2022 and July 2023, with an extension until January 2024. Data analysis included the entire cohort without stratification or comparisons between institutions. Methodological procedures were structured to ensure transparency and reproducibility in accordance with recommended reporting practices for experimental research in educational settings.

Eligible participants were students enrolled in the participating HEIs who had completed coursework in basic anatomy, minor oral surgery, and dental prosthetics and were available to participate in a single research session. All participants provided written informed consent before inclusion. Students with prior knowledge of the lecture topic—either through previous coursework or participation in extension or continuing education activities in implant dentistry—as well as those unable to complete the full session were excluded.

Participants were randomly assigned to one of three groups according to the teaching strategy: group A (theoretical instruction via recorded lecture only), group B (recorded lecture combined with guided laboratory practice), and group C (guided laboratory practice only).

All students initially completed an assessment to measure prior knowledge of the FSS of dental implant placement. This assessment consisted of an objective questionnaire administered via Google Forms comprising five multiple‐choice questions with a single correct answer. The same questionnaire was administered before and after the assigned teaching intervention (pre‐ and posttest design). The instrument used to evaluate students’ perceptions of recorded lectures and hands‐on training was identical across groups, had been tested for reliability, and was aligned with the learning objectives in a prior pilot study involving 10 students.

To ensure equivalence across instructional methods, the recorded lectures and hands‐on sessions were standardized to use the same theoretical framework, learning objectives, and clinical sequence. All assessment questions were developed exclusively from content common to all educational strategies, and no question relied on information presented uniquely in a single instructional method. Additionally, a postintervention questionnaire evaluating students’ perceptions of recorded lectures and hands‐on training was administered to all groups.

The educational activities were conducted in classrooms and dental laboratories using both expository (video‐based lectures) and active, hands‐on methodologies. For the practical activities, an educational tool was developed from a patient‐derived prototype created using clinical examination, dental impressions, and cone‐beam computed tomography (CBCT) images from an anonymized patient who required implant rehabilitation. The prototype reproduced the patient’s anatomical features relevant to implant rehabilitation planning. Written informed consent was obtained from the patient authorizing the use of anonymized clinical and imaging data for the development of educational materials and scientific research.

Based on this patient‐derived prototype, a modified dental mannequin was developed to meet the educational requirements of implant dentistry training. Five industrial silicone molds were subsequently fabricated from the modified mannequin, allowing standardized reproduction of all plaster models used during the educational trial with 115 undergraduate students.

The same instructor delivered the recorded lecture and supervised the practical sessions. The lecture, created using PowerPoint and recorded via Loom (https://www.loom.com), lasted ~15 min and covered the fundamental aspects of the FSS. A recorded format was adopted to minimize presentation bias.

**Figure 1:**
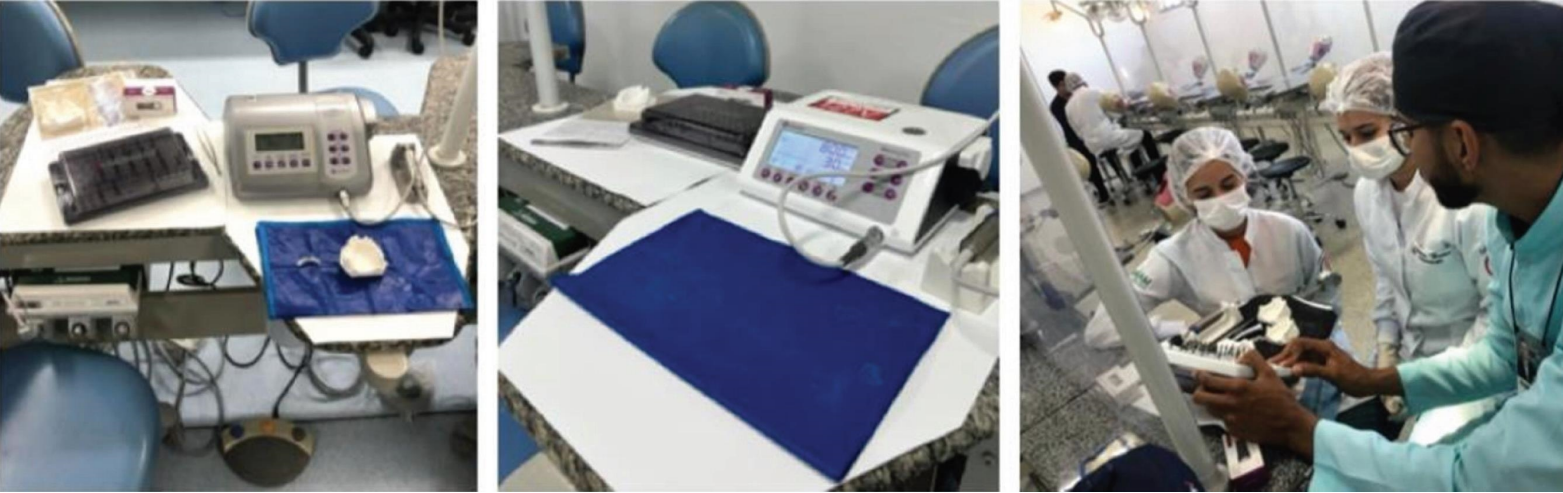
Distribution and composition of the “Boxes” or “Workstations”.

For the practical sessions, four workstations were set up in the laboratory (Figure 1), each supervised by a trained tutor who guided the students and facilitated discussion. Written informed consent was obtained from all identifiable individuals featured in Figure 1 for the publication of their images. Consent was obtained through a signed informed consent form before publication.

**Figure 2:**
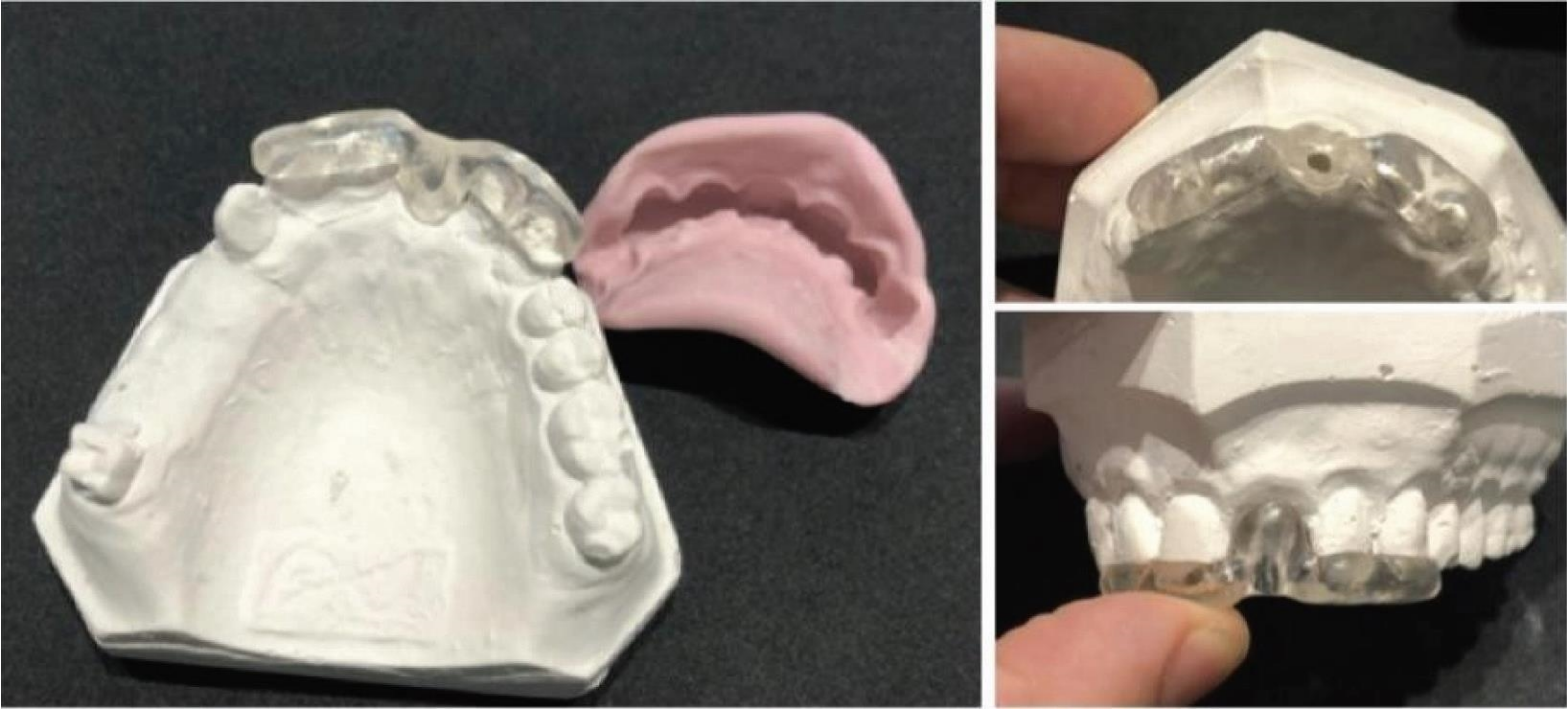
MMA with surgical guide in position.

The educational tool used during the hands‐on activities, referred to as the MAM, consisted of a type II dental plaster model with edentulous areas simulating previous tooth extractions. The clinical scenario showed the absence of tooth 21 (left maxillary central incisor). All MAMs used throughout the study were fabricated from the industrial silicone molds derived from the modified mannequin (Figure 2).

An external prosthetic laboratory fabricated the surgical guides. Eight guides were produced—one for each workstation and an additional spare—and all were standardized and pretested on the models before use. The hands‐on session followed a standardized protocol: (1) hydration of the models by immersion in water for 15–30 min; (2) use of personal protective equipment (PPE); (3) presentation of the master model and materials; (4) discussion of the implant system (Neodent Helix GM, 4.3 mm × 11.5 mm, designated for laboratory training); (5) explanation of the implant motor and drilling kit (one per workstation); (6) presentation of the surgical guide and its role in the FSS; (7) discussion of biological principles and drilling techniques; (8) execution of the drilling sequence according to manufacturer instructions; (9) implant placement following manufacturer guidelines; and (10) placement of the cover screw, followed by final discussion.

At the conclusion of the laboratory activities, a random sample of students—regardless of group allocation—was invited to voluntarily respond to two open‐ended questions via Google Forms (accessed through a QR code) regarding their perceptions of the teaching–learning methodology using the MAM. The questions were as follows: (1) “What was your experience with the applied teaching–learning methodology?” and (2) “If you could describe the activity in one word, what would it be?”

Sample size calculation was performed assuming 90% power, a 5% significance level (*α* = 0.05), and a standard deviation of 0.924 based on pilot data from 12 students. Using a repeated‐measures analysis of variance (ANOVA) model (pre‐ and postintervention) across three groups (A, B, and C), the minimum required sample size was estimated at 23 participants per group to detect a pedagogically relevant difference of at least one correct answer.

All statistical analyses were conducted using R software with a significance level of 5%. Initially, descriptive and exploratory analyses were performed. Variables were summarized using means, standard deviations, medians, minimum and maximum values, and absolute and relative frequencies. ANOVA was used to compare the participant age across groups.

Associations between categorical variables were evaluated using the chi‐square test or Fisher’s exact test as appropriate. Additionally, a generalized linear mixed model for repeated measures was applied to assess the effects of group (A, B, or C) and time (pre‐ and posttest) on the percentage of correct responses. Responses to the open‐ended questions were analyzed using frequency distributions based on the reported perceptions.

## 3. Results

The study population comprised 115 students from four HEIs in Bahia, Brazil: 72 female (62.6%), 42 male (36.5%), and 1 (0.9%) who did not report gender. Students were enrolled in the fifth to tenth semesters of dental school, in accordance with each HEI’s curriculum, to meet the inclusion and exclusion criteria. Accordingly, 22 in the 5th semester (19.2%), 15 in the 6th semester (13.1%), 28 in the 7th semester (24.3%), 7 in the 8th semester (6.0%), 20 in the 9th semester (17.4%), and 23 in the 10th semester (20.0%) were included. Participant ages ranged from 19 to 37 years, with a mean age of 22. No significant differences in sociodemographic characteristics were observed between the groups (*p* > 0.05), as shown in Table 1.

**Table 1:** Comparison results between groups regarding student profiles.

Variable	Group			*p*‐Value
	Recorded lecture	Recorded lecture + hands‐on (practical activity)	Hands‐on (practical activity)	
Sample size	39	39	37	—
Age (years)				
Mean (standard deviation)	22.5 (3.0)	22.0 (2.4)	22.8 (3.4)	0.514
Median (minimum and maximum)	22.0 (19.0; 35.0)	21.0 (19.0; 30.0)	22.0 (20.0; 37.0)	—
Sex, *n* (%)				
Female	27 (69.2%)	23 (59.0%)	22 (59.5%)	0.6248^a^
Male	12 (30.8%)	16 (41.0%)	14 (37.8%)	—
Not reported	0 (0.0%)	0 (0.0%)	1 (2.7%)	—
Undergraduate semester, *n* (%)				
5th	8 (20.5%)	7 (18.0%)	7 (18.9%)	0.9944^a^
6th	4 (10.3%)	6 (15.4%)	5 (13.5%)	—
7th	10 (25.6%)	8 (20.5%)	10 (27.0%)	—
8th	3 (7.7%)	3 (7.7%)	1 (2.7%)	—
9th	6 (15.4%)	8 (20.5%)	6 (16.2%)	—
10th	8 (20.5%)	7 (18.0%)	8 (21.6%)	—

^a^Fisher’s exact test.

**Figure 3:**
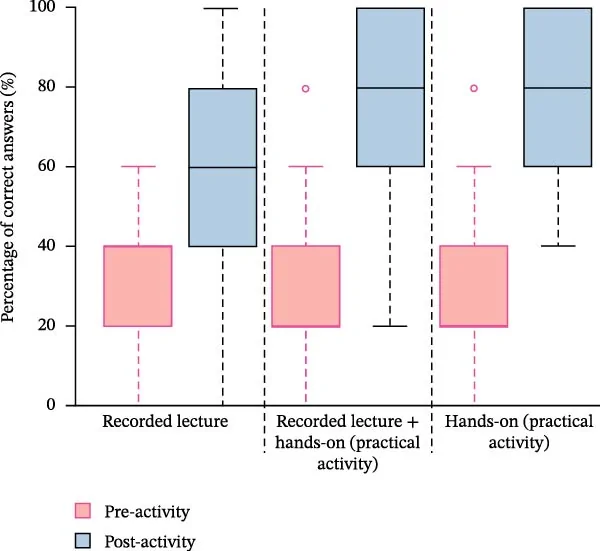
Box plot of the percentage of correct answers to the five questions by group and time.

The results of the analysis of correct answer percentages for the five questions are presented in Table 2 and illustrated in Figure 3. No significant differences were observed between groups in the pretest (*p* > 0.05). A significant increase in correct answer percentages was observed on the posttest across all three groups (*p* < 0.05) compared to the pretest. The percentage of correct answers increased from 33.3% (17.4) to 55.4% (26.1), from 28.2% (20.4) to 76.9% (20.8), and from 23.8% (18.2) to 77.3% (17.7) in groups A, B, and C, respectively. Correct answer percentages were higher in the “Recorded Lecture + hands‐on” (group B) and “Hands‐on only” (group C) groups compared with the “Recorded Lecture” (group A) group (*p* < 0.05). No statistically significant difference was observed between groups B and C.

**Table 2:** Percentage of correct answers to the five questions by group and time.

Group	Period			
	Preactivity		Postactivity	
	Mean (standard deviation)	Median (minimum and maximum)	Mean (standard deviation)	Median (minimum and maximum)
Recorded lesson	33.3 (17.4) Ba	40.0 (0.0; 60.0)	55.4 (26.1) Ab	60.0 (0.0; 100.0)
Recorded lesson + hands‐on	28.2 (20.4) Ba	20.0 (0.0; 80.0)	76.9 (20.8) Aa	80.0 (20.0; 100.0)
Hands‐on	23.8 (18.2) Ba	20.0 (0.0; 80.0)	77.3 (17.7) Aa	80.0 (40.0; 100.0)

*Note: p* (group) = 0.4199; *p* (time) < 0.0001; *p* (interaction) = 0.0241. Different letters (capital letters indicating horizontal comparisons of assessment times and lowercase letters indicating vertical comparisons of groups) indicate statistically significant differences (*p* ≤ 0.05).

Regarding the subjective questions, 89 students answered question 1 and 60 answered question 2, representing 77.4% and 52.1% of participants, respectively. For the first question, “How was your experience with the applied methodology or the teaching–learning process?” (Table 3), nearly all participants (99.2%) preferred the applied methodology over the traditional recorded lecture. Responses were grouped into central themes for analysis. Students’ perceptions and experiences regarding the applied teaching methodology were quantitatively categorized by thematic frequency and are presented in Tables 3 and 4.

**Table 3:** Students’ perceptions of the applied teaching methodology.

Core perception category	Percentage (%)
Preference for the applied methodology compared to the recorded lecture	99.2
Direct and immediate association between theory and practice	31.9
Access to relevant global clinical dental content during undergraduate training	11.0
Motivation in the teaching–learning process	11.0
Memorable academic experience	9.5
Praise, gratitude, and desire for similar future experiences	7.7
Objectivity of discussion	6.0
Innovative methodology	4.3
Playful approach	3.5
Dynamic methodology	2.6
Tutors’ confidence and expertise	2.6

**Table 4:** Summary of the methodology described in one word by the students.

Word/concept	Percentage (%)
“Wonderful” and similar adjectives	43.9
Didactically efficient for learning	30.7
Enriching	8.3
Motivating	6.8
Innovative	5.0
Dynamic	3.4
“Only those who lack interest fail to understand and learn”	1.7

The main perceptual theme identified from the responses was the direct and immediate integration of theory and practice or even theorizing from practice, which was the most frequently mentioned element (31.9%). The opportunity to access clinically relevant content in implant dentistry as an undergraduate accounted for 11% of responses, as did increased motivation associated with the teaching–learning process (11%).

Students described the activity as “remarkable” in 9.5% of responses, often citing the discussion’s objectivity (6%). The MMA was also characterized as innovative (4.3%), playful (3.5%), and dynamic (2.6%). Tutor support was highlighted as a relevant factor by 2.6% of participants. Additional responses expressing satisfaction, gratitude, or interest in similar experiences accounted for 7.7%.

For the question, “If you could describe the applied teaching methodology in just one word, what would it be?” (Table 4), the term “wonderful” and similar adjectives accounted for 43.9% of responses. Descriptions related to effective learning accounted for 30.7% of the total, while 8.3% described the methodology as enriching. Other responses included motivating (6.8%), innovative (5.0%), and dynamic (3.4%). One participant (1.7%) provided a qualitative statement emphasizing that learning depends on student engagement.

## 4. Discussion

Analysis of the results showed that student groups were successfully randomized with respect to gender, academic semester, and age (*p* > 0.05). The vast majority of students were young, with a mean age of 22, corroborating national data [14, 15]. When considering knowledge gains across the different teaching–learning modalities, correct answers increased significantly (*p* < 0.05) in the pre‐ and posttests for all modalities, indicating improved cognitive learning. However, theoretical instruction (group A) contributed the least, with an increase of 22.1%, compared to 48.9% for theoretical instruction followed by guided practice (group B) and 53.5% for guided practice only (group C) [16, 17].

Students exposed to ALMs achieved 2.2–2.4 times as many correct answers as those receiving only theoretical instruction, with slightly higher values for guided practice alone. However, no statistically significant difference was observed between the ALMs (*p* > 0.05). These findings suggest that a theoretical lecture preceding the practical activity did not provide additional cognitive benefit for the analyzed content—the scarcity of comparable quantitative research [2] limits comparisons with other studies.

Most studies directly related to this topic are descriptive and qualitative, primarily focusing on student perceptions assessed through interviews or questionnaires [18, 19]. In the present study, analysis of open‐ended responses identified key themes and 116 positive elements, with an acceptance rate of ~98.3%. Given that one negative comment addressed the pretest rather than the methodology itself and that two participants provided constructive suggestions, the overall acceptance can be considered extremely high. Previous studies report satisfaction rates of 98%–100%, while rates as low as 56% are already considered high [20–22].

Findings from Tables 3 and 4 indicate that hands‐on experience in implant dentistry is associated with increased perceived technical competence, improved understanding of biomechanical principles, and greater self‐confidence in clinical practice. Similar results were reported by Ziada et al. [23], who demonstrated that simulated activities enhance psychomotor development and clinical reasoning by integrating theory and practice. Participants also reported improved understanding of surgical steps and three‐dimensional implant positioning following hands‐on training [23].

The high level of agreement (Table 3) regarding spatial understanding of implant placement is consistent with findings by Al‐Saud et al. [24], who reported that simulated environments facilitate anatomical integration, refinement of motor skills, and deeper cognitive processing compared to traditional lecture‐based teaching.

Similarly, increased confidence for future clinical interventions reinforces the evidence that hands‐on experience reduces anxiety and supports the transition to patient care (Table 4) [25]. Self‐confidence has been described as a mediating factor between simulation‐based training and clinical performance [26]. Furthermore, structured formative feedback during simulation enhances skill consolidation and reflective learning [27]. These findings support the integration of supervised practice and feedback as key components in the development of both technical competence and professional identity.

Dental education is inherently practice‐oriented, linking theory and clinical application. Thus, active engagement through “learning by doing” is fundamental not only for psychomotor development but also for meaningful knowledge construction [8].

The present findings also support the concept of “theorizing from practice” [28]. This may explain group C’s performance, in which students with limited prior knowledge understood and performed the activity without prior theoretical instruction. These results suggest that the traditional sequence of theory preceding practice may not always be necessary and that, in some contexts, practice‐first or integrated approaches may enhance learning. Such methodological flexibility supports the view of learning as a dynamic and adaptive process [7, 11, 22].

Although ALMs, including hands‐on training, offer clear advantages, they do not replace traditional lectures. Rather, a balanced approach that integrates theoretical instruction with supervised practical experience is essential for developing comprehensive and confident clinical competence [29, 30].

Technological resources, including simulation laboratories and information and communication technologies, can further enhance learning [31]. However, this study demonstrates that effective training can also be achieved with low‐cost, reproducible tools, such as the MMA, which was well accepted and effective [20].

Although ALMs require additional planning, tutor supervision, and laboratory resources, the MMA was specifically designed to be a low‐cost, reusable educational tool. The use of reusable plaster models, small rotating groups, and asynchronous recorded content may optimize institutional resources while maintaining educational effectiveness. These findings are consistent with studies demonstrating that simulation‐based education improves psychomotor development, spatial understanding, clinical confidence, and patient safety while reducing future procedural complications. Hybrid educational models integrating practical sessions with digital resources have also been proposed as cost‐effective approaches to implant dentistry education [28, 32].

The similarity in posttest performance between groups B and C suggests that recorded lectures may not significantly improve short‐term cognitive outcomes when compared with supervised practical training alone. However, recorded lectures remain pedagogically valuable because they offer flexibility, self‐paced review opportunities, and reinforcement of theoretical concepts, particularly for students who require additional study time [3, 28]. Student perception data further demonstrated high satisfaction with hands‐on methodologies, emphasizing motivation, confidence, and the integration of theory and practice as relevant factors for future curriculum development in implant dentistry [17, 23, 24].

Hands‐on methodologies, although more resource‐intensive, are strongly supported by the literature. Repeated practice combined with structured feedback leads to significant improvements in precision, spatial control, and skill retention during implant‐related procedures [33, 34]. These findings align with the principles of deliberate practice, which hold that guided repetition and immediate feedback are essential for long‐term skill acquisition [35].

Beyond technical benefits, simulation‐based training has important implications for patient safety by reducing errors during the transition to clinical practice [36]. From an economic perspective, the reduction in complications may offset the initial investment required for implementing such methodologies. Hybrid models combining digital learning with practical sessions have also been proposed to optimize resources while maintaining the educational quality [28].

Soni et al. [37], in their analysis of simulation‐based teaching programs, highlight that implementing these strategies must necessarily be accompanied by specific training for teachers and monitors responsible for conducting practical activities. According to the authors, this structured qualification of simulation resources reduces the direct teaching load, improves the pedagogical quality of practical sessions, promotes greater efficiency in the use of laboratory time, optimizes institutional resources, and expands the capacity for student supervision while maintaining the technical benefits of hands‐on learning [37]. Given this scenario, the present study reinforces the pedagogical benefits of active methodologies. The authors suggest that implementing this methodology in the teaching–learning process within dentistry curricula can facilitate the prediction of required financial investment and thus enable more rational planning for infrastructure and continuing professional development expenses.

Student satisfaction and clinical readiness are also positively influenced by structured practical training. Previous studies have demonstrated increased engagement, confidence, and accelerated learning among students who have been exposed to guided, hands‐on experiences [38, 39].

Another relevant aspect concerns student satisfaction and clinical readiness with this type of active methodology. Shetty et al. [38] and Fischer et al. [39] evaluated structured practical courses that included guided planning and supervised execution and demonstrated increased confidence, greater engagement, and an accelerated learning curve among students. These factors are particularly important in implant dentistry, an area that requires the integration of theoretical knowledge, diagnostic reasoning, and refined psychomotor skills [38, 39].

Learning effectiveness appears to result from a combination of interacting factors, including methodology, the environment, and student engagement. The learning environment created in this study likely contributed to the positive perceptions reported [21].

Ziada et al. [23] qualitatively analyzed students’ reflections on practical training in implantology. They demonstrated that this active teaching methodology not only consolidates theoretical knowledge but also improves essential psychomotor skills, such as instrumental manipulation, three‐dimensional implant positioning, and control of the surgical sequence. In line with these findings, Mohammed et al. [40] identified gaps in clinical training in implantology, highlighting that isolated conceptual mastery does not guarantee complete professional competence. The authors recommend the adoption of curricula that contemplate the integrated development of cognitive skills (reverse planning and evidence‐based decision‐making), psychomotor skills (simulated and supervised surgical execution), clinical skills (development of treatment plans and initial management of complications), and behavioral skills (teamwork, communication, and professional ethics). Thus, the systematic inclusion of supervised practical training in the curriculum, which complements the traditional lecture format, is an essential strategy to ensure high‐quality technical training, patient safety, and alignment with the contemporary demands of the implant dentistry practice.

The relevance and contemporary nature of the research subject, coupled with students’ voluntary participation, may explain their remarkably positive comments and perceptions. It is important to note that this situation likely differs from typical classrooms, where students with varied aptitudes and interests are required to participate in all activities, often discussing less engaging content, which can potentially influence learning levels [11, 16, 21].

It is also noteworthy that the ALMs using MMAs required considerably more time and a greater number of tutors for content discussion than for lectures, as well as more technical resources, equipment, strategic planning, and overall coordination of activities.

Furthermore, to encourage discussion and knowledge development, content and class sizes must be reduced, and more time must be allocated to this process [1, 9]. Only basic or foundational concepts of the topic should be discussed, with later outside‐the‐classroom activities such as seminars, recorded lessons, quizzes, and directed studies [13].

Unlike lectures that require only one teacher, the MMA application required a team, including trainees and monitors who were trained as tutors. The maximum number of students per session was limited to 36, divided into three groups of 12. For problem‐based learning, for example, teachers need to work with groups of 10–12 students, a group size similar to that used here [6].

The student perceptions identified in this study provide relevant insights for future planning of implant dentistry curricula. The high levels of satisfaction, motivation, and self‐confidence reported by participants reinforce the importance of integrating supervised hands‐on methodologies with complementary theoretical resources. These findings suggest that hybrid curricula combining active learning, clinical simulation, and asynchronous digital content may promote a more efficient, student‐centered teaching–learning process aligned with contemporary demands in dental education [28, 29, 32]. Furthermore, students’ appreciation of practical experience demonstrates that interactive learning environments may contribute not only to cognitive and psychomotor development but also to strengthening clinical confidence and future professional preparedness [23, 24, 38, 39].

Finally, the main limitation of this study is that only cognitive outcomes were assessed. Nevertheless, the findings demonstrate that MMA is an effective and practical tool for promoting cognitive learning. Future studies should explore its impact on psychomotor and clinical performance as well as the integration of digital simulation technologies.

## 5. Conclusion

The results of this study confirmed the effectiveness of MMA in developing cognitive competencies related to the FSS in implant dentistry. Using the MMA tool to teach FSS was twice as effective as theoretical instruction alone in promoting cognitive learning. Furthermore, the integration between theory and practice was the element most frequently perceived by students, contributing to the high success rate of the applied ALM; maintaining this sequence or including prior theoretical instruction was not essential.

## Funding

The research did not receive any specific funding.

## Disclosure

After using Grammarly, the authors reviewed and edited the content as necessary and take full responsibility for the publication’s content. The authors also state that this research was part of a doctoral thesis, which is available at https://repositorio.bahiana.edu.br/server/api/core/bitstreams/168d0b2f‐2f05‐4be4‐a409‐a819f78cb91e/content.

## Conflicts of Interest

The authors declare no conflicts of interest.

## Data Availability

The data that support the findings of this study are available upon request from the corresponding author. The data are not publicly available due to privacy or ethical restrictions.
